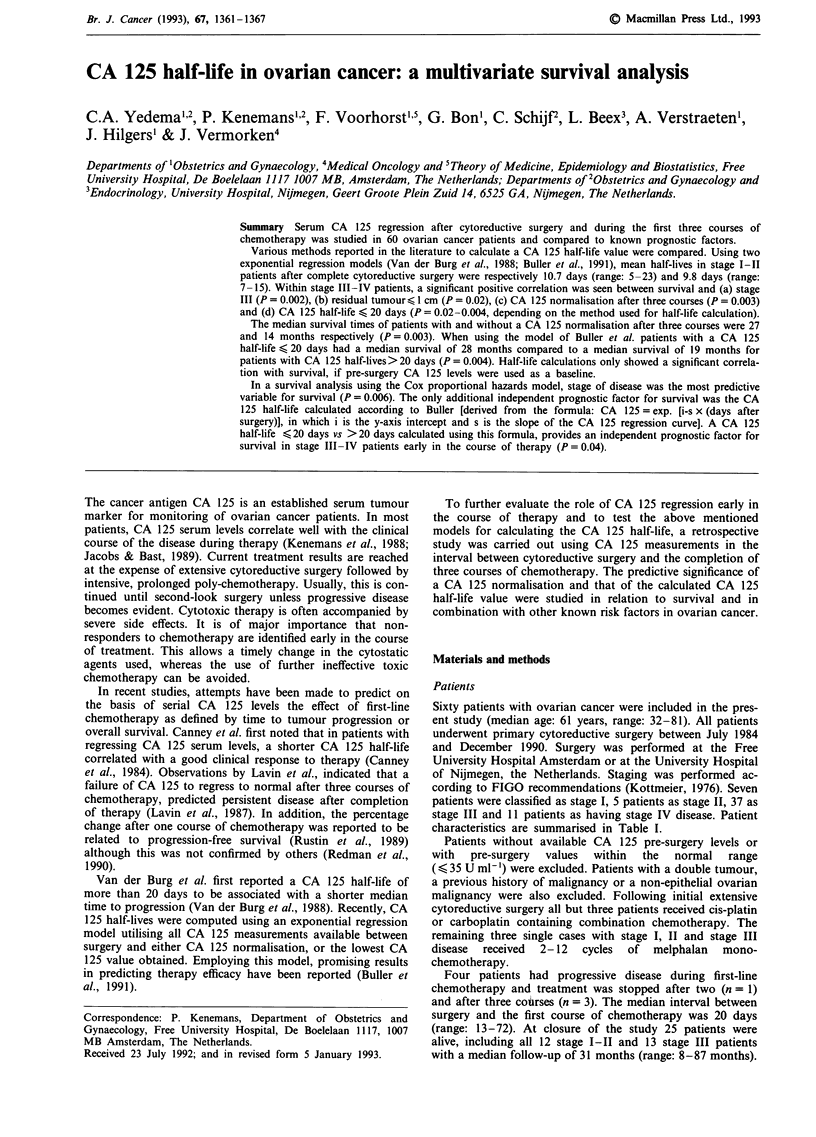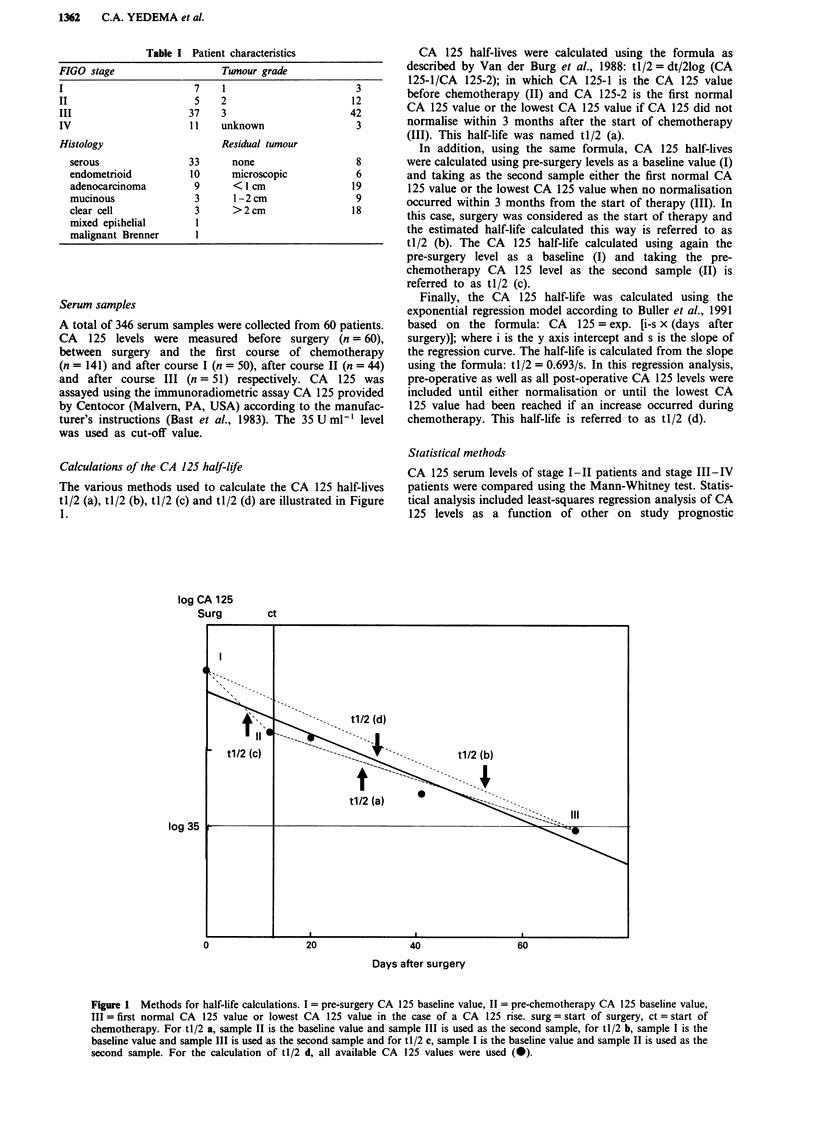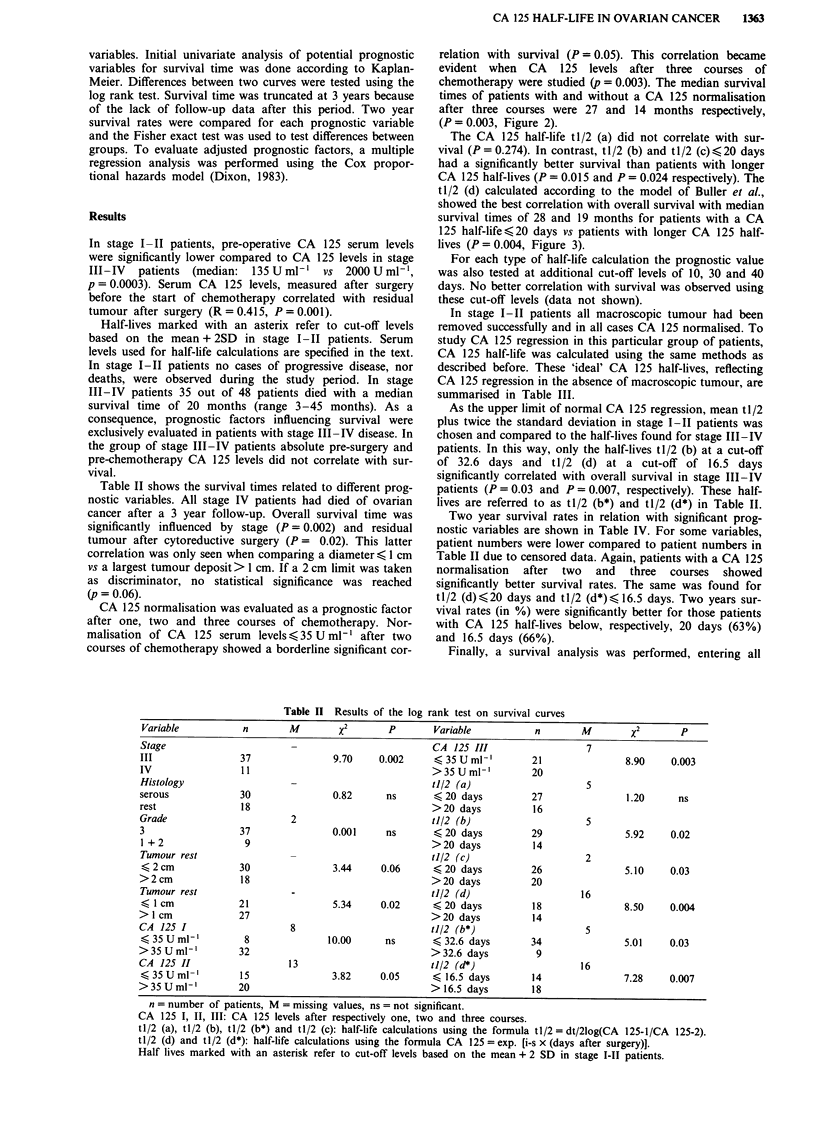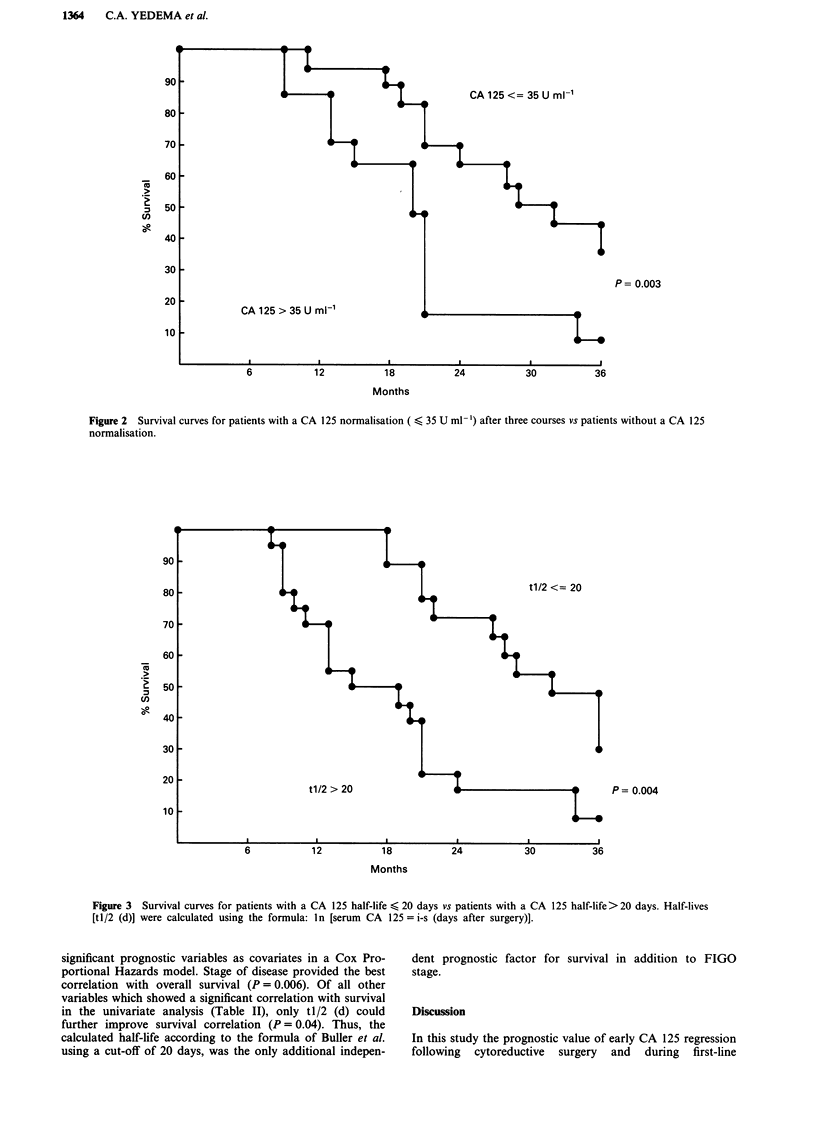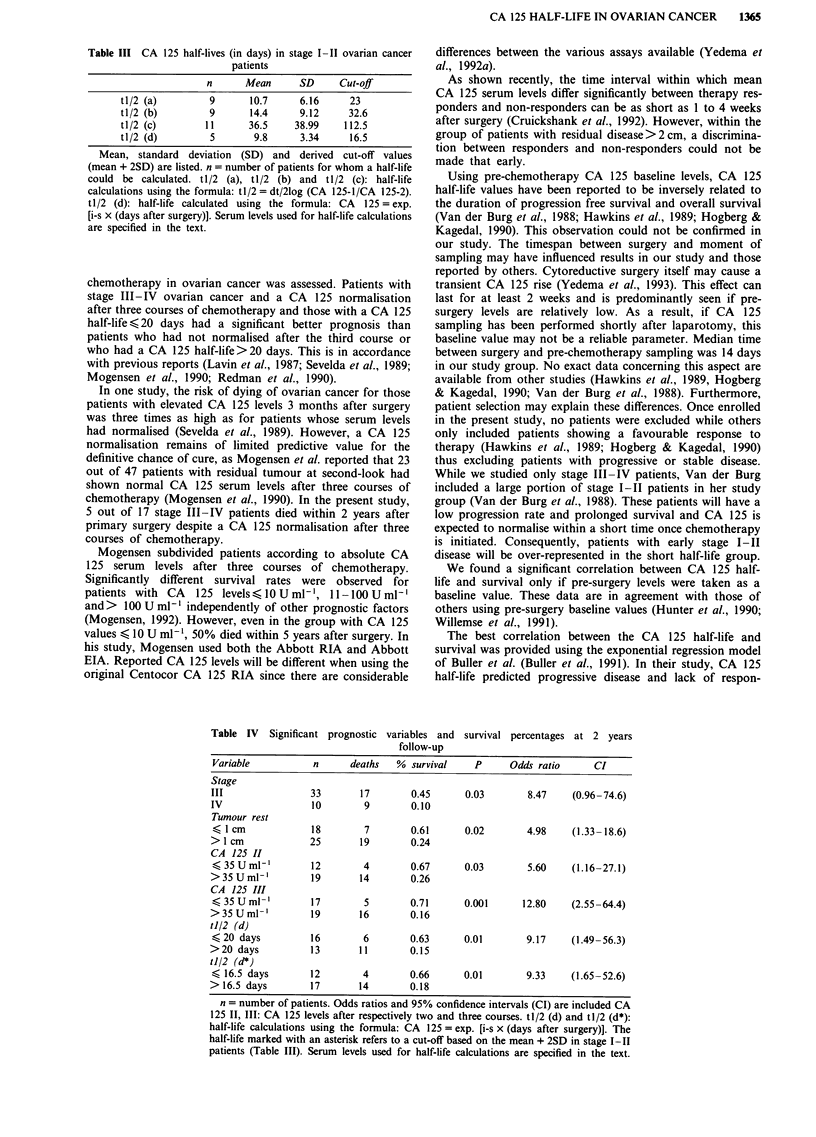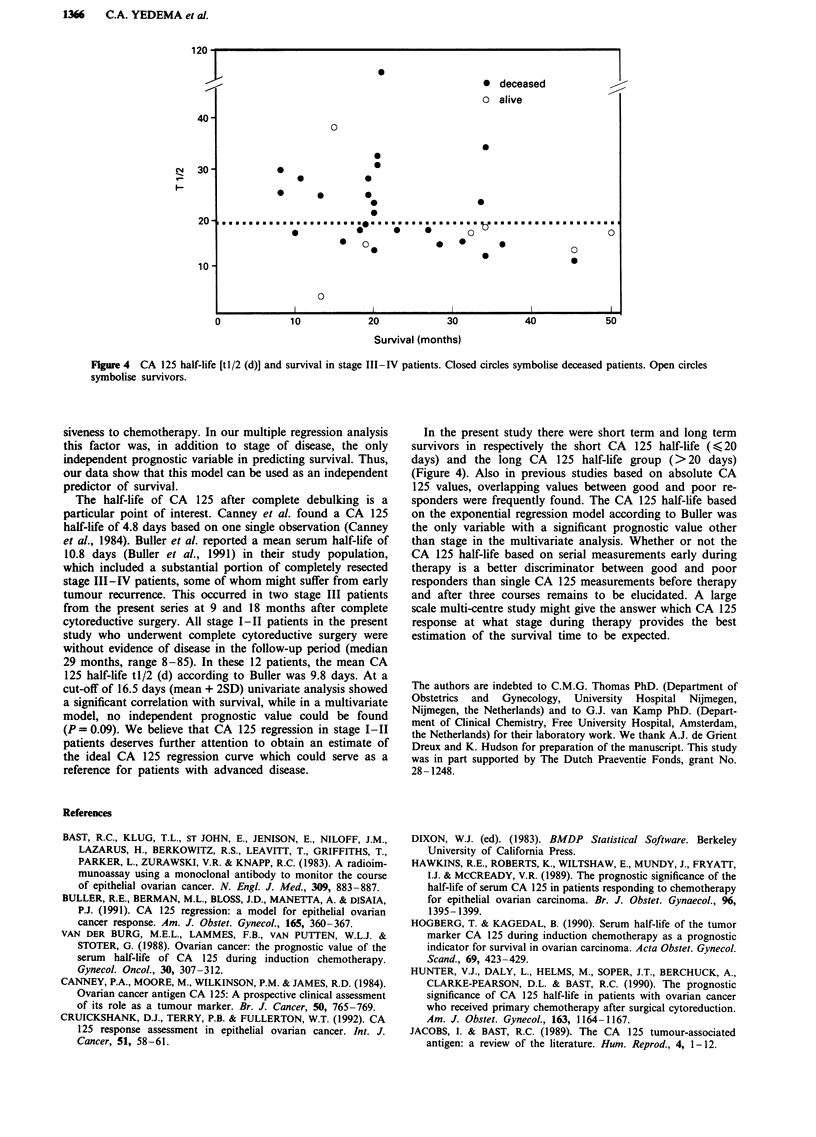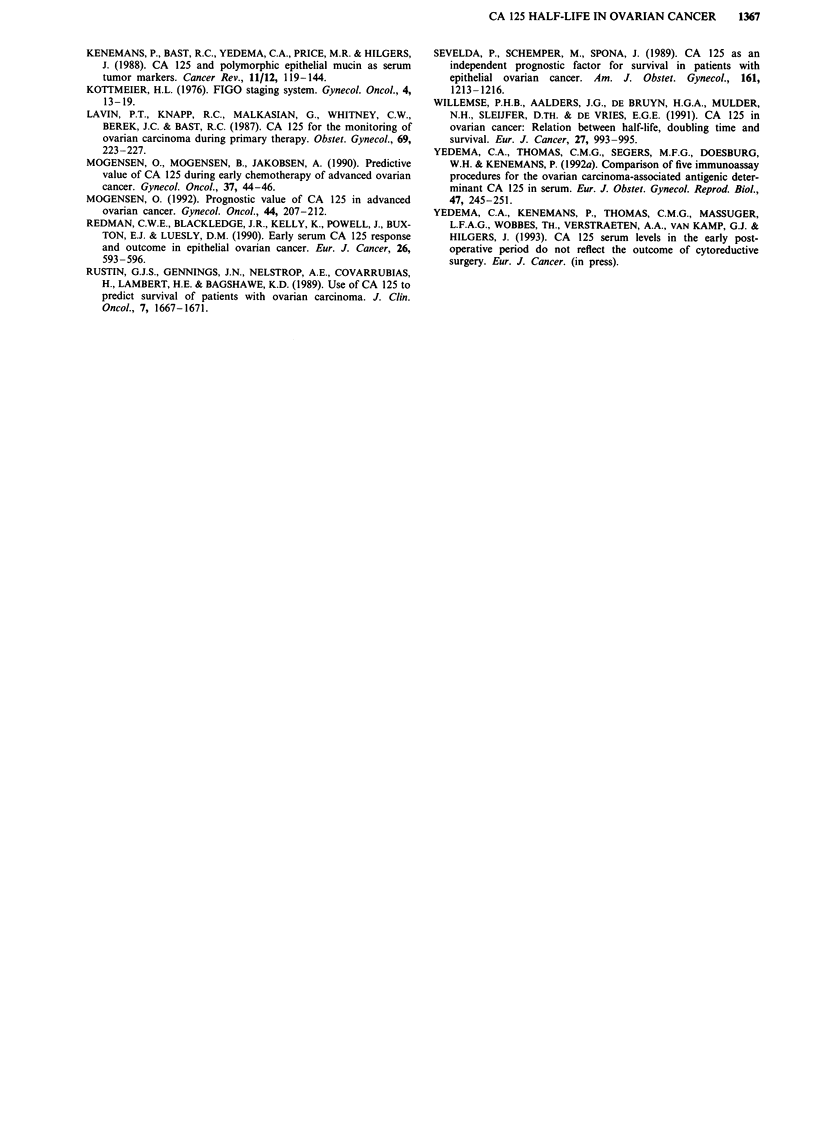# CA 125 half-life in ovarian cancer: a multivariate survival analysis.

**DOI:** 10.1038/bjc.1993.252

**Published:** 1993-06

**Authors:** C. A. Yedema, P. Kenemans, F. Voorhorst, G. Bon, C. Schijf, L. Beex, A. Verstraeten, J. Hilgers, J. Vermorken

**Affiliations:** Department of Obstetrics and Gynaecology, Free University Hospital, Amsterdam, The Netherlands.

## Abstract

Serum CA 125 regression after cytoreductive surgery and during the first three courses of chemotherapy was studied in 60 ovarian cancer patients and compared to known prognostic factors. Various methods reported in the literature to calculate a CA 125 half-live value were compared. Using two exponential regression models (Van der Burg et al., 1988; Buller et al., 1991), mean half-lives in stage I-II patients after complete cytoreductive surgery were respectively 10.7 days (range: 5-23) and 9.8 days (range: 7-15). Within stage III-IV patients, a significant positive correlation was seen between survival and (a) stage III (P = 0.002), (b) residual tumour < or = 1 cm (P = 0.02), (c) CA 125 normalisation after three courses (P = 0.003) and (d) CA 125 half-life < or = 20 days (P = 0.02-0.004, depending on the method used for half-life calculation). The median survival times of patients with and without a CA 125 normalisation after three courses were 27 and 14 months respectively (P = 0.003). When using the model of Buller et al. patients with a CA 125 half-life < or = 20 days had a median survival of 28 months compared to a median survival of 19 months for patients with CA 125 half-lives > 20 days (P = 0.004). Half-life calculations only showed a significant correlation with survival, if pre-surgery CA 125 levels were used as a baseline. In a survival analysis using the Cox proportional hazards model, stage of disease was the most predictive variable for survival (P = 0.006). The only additional independent prognostic factor for survival was the CA 125 half-life calculated according to Buller [derived from the formula: CA 125 = exp. [i-s x (days after surgery)], in which i is the y-axis intercept and s is the slope of the CA 125 regression curve]. A CA 125 half-life < or = 20 days vs > 20 days calculated using this formula, provides an independent prognostic factor for survival in stage III-IV patients early in the course of therapy (P = 0.04).


Br. J. Cancer (1993), 67, 1361–1367 © Macmillan Press Ltd., 1993

### CA 125 half-life in ovarian cancer: a multivariate survival analysis

C.A. Yedema[1,2], P. Kenemans[1,2], F. Voorhorst[1,5], G. Bon[1], C. Schijf[2], L. Beex[3], A. Verstraeten[1], J. Hilgers[1] & J. Vermorken[4]

*Departments of [1]Obstetrics and Gynaecology, [4]Medical Oncology and [5]Theory of Medicine, Epidemiology and Biostatistics, Free University Hospital, De Boelelaan 1117 1007 MB, Amsterdam, The Netherlands; Departments of [2]Obstetrics and Gynaecology and [3]Endocrinology, University Hospital, Nijmegen, Geert Groote Plein Zuid 14, 6525 GA, Nijmegen, The Netherlands.*

**Summary** Serum CA 125 regression after cytoreductive surgery and during the first three courses of chemotherapy was studied in 60 ovarian cancer patients and compared to known prognostic factors.

Various methods reported in the literature to calculate a CA 125 half-life value were compared. Using two exponential regression models (Van der Burg *et al.*, 1988; Buller *et al.*, 1991), mean half-lives in stage I–II patients after complete cytoreductive surgery were respectively 10.7 days (range: 5–23) and 9.8 days (range: 7–15). Within stage III–IV patients, a significant positive correlation was seen between survival and (a) stage III ($P = 0.002$), (b) residual tumour $\leqslant$ 1 cm ($P = 0.02$), (c) CA 125 normalisation after three courses ($P = 0.003$) and (d) CA 125 half-life $\leqslant$ 20 days ($P = 0.02$–$0.004$, depending on the method used for half-life calculation).

The median survival times of patients with and without a CA 125 normalisation after three courses were 27 and 14 months respectively ($P = 0.003$). When using the model of Buller *et al.* patients with a CA 125 half-life $\leqslant$ 20 days had a median survival of 28 months compared to a median survival of 19 months for patients with CA 125 half-lives $>$ 20 days ($P = 0.004$). Half-life calculations only showed a significant correlation with survival, if pre-surgery CA 125 levels were used as a baseline.

In a survival analysis using the Cox proportional hazards model, stage of disease was the most predictive variable for survival ($P = 0.006$). The only additional independent prognostic factor for survival was the CA 125 half-life calculated according to Buller [derived from the formula: CA 125 = exp. [i-s × (days after surgery)], in which i is the y-axis intercept and s is the slope of the CA 125 regression curve]. A CA 125 half-life $\leqslant$ 20 days *vs* $>$ 20 days calculated using this formula, provides an independent prognostic factor for survival in stage III–IV patients early in the course of therapy ($P = 0.04$).

The cancer antigen CA 125 is an established serum tumour marker for monitoring of ovarian cancer patients. In most patients, CA 125 serum levels correlate well with the clinical course of the disease during therapy (Kenemans *et al.*, 1988; Jacobs & Bast, 1989). Current treatment results are reached at the expense of extensive cytoreductive surgery followed by intensive, prolonged poly-chemotherapy. Usually, this is continued until second-look surgery unless progressive disease becomes evident. Cytotoxic therapy is often accompanied by severe side effects. It is of major importance that non-responders to chemotherapy are identified early in the course of treatment. This allows a timely change in the cytostatic agents used, whereas the use of further ineffective toxic chemotherapy can be avoided.

In recent studies, attempts have been made to predict on the basis of serial CA 125 levels the effect of first-line chemotherapy as defined by time to tumour progression or overall survival. Canney *et al.* first noted that in patients with regressing CA 125 serum levels, a shorter CA 125 half-life correlated with a good clinical response to therapy (Canney *et al.*, 1984). Observations by Lavin *et al.*, indicated that a failure of CA 125 to regress to normal after three courses of chemotherapy, predicted persistent disease after completion of therapy (Lavin *et al.*, 1987). In addition, the percentage change after one course of chemotherapy was reported to be related to progression-free survival (Rustin *et al.*, 1989) although this was not confirmed by others (Redman *et al.*, 1990).

Van der Burg *et al.* first reported a CA 125 half-life of more than 20 days to be associated with a shorter median time to progression (Van der Burg *et al.*, 1988). Recently, CA 125 half-lives were computed using an exponential regression model utilising all CA 125 measurements available between surgery and either CA 125 normalisation, or the lowest CA 125 value obtained. Employing this model, promising results in predicting therapy efficacy have been reported (Buller *et al.*, 1991).

Correspondence: P. Kenemans, Department of Obstetrics and Gynaecology, Free University Hospital, De Boelelaan 1117, 1007 MB Amsterdam, The Netherlands.
Received 23 July 1992; and in revised form 5 January 1993.

To further evaluate the role of CA 125 regression early in the course of therapy and to test the above mentioned models for calculating the CA 125 half-life, a retrospective study was carried out using CA 125 measurements in the interval between cytoreductive surgery and the completion of three courses of chemotherapy. The predictive significance of a CA 125 normalisation and that of the calculated CA 125 half-life value were studied in relation to survival and in combination with other known risk factors in ovarian cancer.

### Materials and methods

### *Patients*

Sixty patients with ovarian cancer were included in the present study (median age: 61 years, range: 32–81). All patients underwent primary cytoreductive surgery between July 1984 and December 1990. Surgery was performed at the Free University Hospital Amsterdam or at the University Hospital of Nijmegen, the Netherlands. Staging was performed according to FIGO recommendations (Kottmeier, 1976). Seven patients were classified as stage I, 5 patients as stage II, 37 as stage III and 11 patients as having stage IV disease. Patient characteristics are summarised in Table I.

Patients without available CA 125 pre-surgery levels or with pre-surgery values within the normal range ($\leqslant$35 U ml$^{-1}$) were excluded. Patients with a double tumour, a previous history of malignancy or a non-epithelial ovarian malignancy were also excluded. Following initial extensive cytoreductive surgery all but three patients received cis-platin or carboplatin containing combination chemotherapy. The remaining three single cases with stage I, II and stage III disease received 2–12 cycles of melphalan mono-chemotherapy.

Four patients had progressive disease during first-line chemotherapy and treatment was stopped after two ($n = 1$) and after three courses ($n = 3$). The median interval between surgery and the first course of chemotherapy was 20 days (range: 13–72). At closure of the study 25 patients were alive, including all 12 stage I–II and 13 stage III patients with a median follow-up of 31 months (range: 8–87 months).

Table I Patient characteristics

| *FIGO stage* | | *Tumour grade* | |
|---|---|---|---|
| I | 7 | 1 | 3 |
| II | 5 | 2 | 12 |
| III | 37 | 3 | 42 |
| IV | 11 | unknown | 3 |
| *Histology* | | *Residual tumour* | |
| serous | 33 | none | 8 |
| endometrioid | 10 | microscopic | 6 |
| adenocarcinoma | 9 | <1 cm | 19 |
| mucinous | 3 | 1-2 cm | 9 |
| clear cell | 3 | >2 cm | 18 |
| mixed epithelial | 1 | | |
| malignant Brenner | 1 | | |

### *Serum samples*

A total of 346 serum samples were collected from 60 patients. CA 125 levels were measured before surgery ($n = 60$), between surgery and the first course of chemotherapy ($n = 141$) and after course I ($n = 50$), after course II ($n = 44$) and after course III ($n = 51$) respectively. CA 125 was assayed using the immunoradiometric assay CA 125 provided by Centocor (Malvern, PA, USA) according to the manufacturer's instructions (Bast *et al.*, 1983). The 35 U $ml^{-1}$ level was used as cut-off value.

### *Calculations of the CA 125 half-life*

The various methods used to calculate the CA 125 half-lives t1/2 (a), t1/2 (b), t1/2 (c) and t1/2 (d) are illustrated in Figure 1.

CA 125 half-lives were calculated using the formula as described by Van der Burg *et al.*, 1988: t1/2 = dt/2log (CA 125-1/CA 125-2); in which CA 125-1 is the CA 125 value before chemotherapy (II) and CA 125-2 is the first normal CA 125 value or the lowest CA 125 value if CA 125 did not normalise within 3 months after the start of chemotherapy (III). This half-life was named t1/2 (a).

In addition, using the same formula, CA 125 half-lives were calculated using pre-surgery levels as a baseline value (I) and taking as the second sample either the first normal CA 125 value or the lowest CA 125 value when no normalisation occurred within 3 months from the start of therapy (III). In this case, surgery was considered as the start of therapy and the estimated half-life calculated this way is referred to as t1/2 (b). The CA 125 half-life calculated using again the pre-surgery level as a baseline (I) and taking the pre-chemotherapy CA 125 level as the second sample (II) is referred to as t1/2 (c).

Finally, the CA 125 half-life was calculated using the exponential regression model according to Buller *et al.*, 1991 based on the formula: CA 125 = exp. [i-s × (days after surgery)]; where i is the y axis intercept and s is the slope of the regression curve. The half-life is calculated from the slope using the formula: t1/2 = 0.693/s. In this regression analysis, pre-operative as well as all post-operative CA 125 levels were included until either normalisation or until the lowest CA 125 value had been reached if an increase occurred during chemotherapy. This half-life is referred to as t1/2 (d).

### *Statistical methods*

CA 125 serum levels of stage I-II patients and stage III-IV patients were compared using the Mann-Whitney test. Statistical analysis included least-squares regression analysis of CA 125 levels as a function of other on study prognostic

**Figure 1** Methods for half-life calculations. I = pre-surgery CA 125 baseline value, II = pre-chemotherapy CA 125 baseline value, III = first normal CA 125 value or lowest CA 125 value in the case of a CA 125 rise. surg = start of surgery, ct = start of chemotherapy. For t1/2 **a**, sample II is the baseline value and sample III is used as the second sample, for t1/2 **b**, sample I is the baseline value and sample III is used as the second sample and for t1/2 **c**, sample I is the baseline value and sample II is used as the second sample. For the calculation of t1/2 **d**, all available CA 125 values were used (●).

variables. Initial univariate analysis of potential prognostic variables for survival time was done according to Kaplan-Meier. Differences between two curves were tested using the log rank test. Survival time was truncated at 3 years because of the lack of follow-up data after this period. Two year survival rates were compared for each prognostic variable and the Fisher exact test was used to test differences between groups. To evaluate adjusted prognostic factors, a multiple regression analysis was performed using the Cox proportional hazards model (Dixon, 1983).

### Results

In stage I–II patients, pre-operative CA 125 serum levels were significantly lower compared to CA 125 levels in stage III–IV patients (median: $135\,U\,ml^{-1}$ *vs* $2000\,U\,ml^{-1}$, $p = 0.0003$). Serum CA 125 levels, measured after surgery before the start of chemotherapy correlated with residual tumour after surgery ($R = 0.415$, $P = 0.001$).

Half-lives marked with an asterix refer to cut-off levels based on the mean + 2SD in stage I–II patients. Serum levels used for half-life calculations are specified in the text. In stage I–II patients no cases of progressive disease, nor deaths, were observed during the study period. In stage III–IV patients 35 out of 48 patients died with a median survival time of 20 months (range 3–45 months). As a consequence, prognostic factors influencing survival were exclusively evaluated in patients with stage III–IV disease. In the group of stage III–IV patients absolute pre-surgery and pre-chemotherapy CA 125 levels did not correlate with survival.

Table II shows the survival times related to different prognostic variables. All stage IV patients had died of ovarian cancer after a 3 year follow-up. Overall survival time was significantly influenced by stage ($P = 0.002$) and residual tumour after cytoreductive surgery ($P = 0.02$). This latter correlation was only seen when comparing a diameter ⩽ 1 cm *vs* a largest tumour deposit > 1 cm. If a 2 cm limit was taken as discriminator, no statistical significance was reached ($p = 0.06$).

CA 125 normalisation was evaluated as a prognostic factor after one, two and three courses of chemotherapy. Normalisation of CA 125 serum levels ⩽ $35\,U\,ml^{-1}$ after two courses of chemotherapy showed a borderline significant correlation with survival ($P = 0.05$). This correlation became evident when CA 125 levels after three courses of chemotherapy were studied ($p = 0.003$). The median survival times of patients with and without a CA 125 normalisation after three courses were 27 and 14 months respectively, ($P = 0.003$, Figure 2).

The CA 125 half-life t1/2 (a) did not correlate with survival ($P = 0.274$). In contrast, t1/2 (b) and t1/2 (c) ⩽ 20 days had a significantly better survival than patients with longer CA 125 half-lives ($P = 0.015$ and $P = 0.024$ respectively). The t1/2 (d) calculated according to the model of Buller *et al.*, showed the best correlation with overall survival with median survival times of 28 and 19 months for patients with a CA 125 half-life ⩽ 20 days *vs* patients with longer CA 125 half-lives ($P = 0.004$, Figure 3).

For each type of half-life calculation the prognostic value was also tested at additional cut-off levels of 10, 30 and 40 days. No better correlation with survival was observed using these cut-off levels (data not shown).

In stage I–II patients all macroscopic tumour had been removed successfully and in all cases CA 125 normalised. To study CA 125 regression in this particular group of patients, CA 125 half-life was calculated using the same methods as described before. These 'ideal' CA 125 half-lives, reflecting CA 125 regression in the absence of macroscopic tumour, are summarised in Table III.

As the upper limit of normal CA 125 regression, mean t1/2 plus twice the standard deviation in stage I–II patients was chosen and compared to the half-lives found for stage III–IV patients. In this way, only the half-lives t1/2 (b) at a cut-off of 32.6 days and t1/2 (d) at a cut-off of 16.5 days significantly correlated with overall survival in stage III–IV patients ($P = 0.03$ and $P = 0.007$, respectively). These half-lives are referred to as t1/2 (b*) and t1/2 (d*) in Table II.

Two year survival rates in relation with significant prognostic variables are shown in Table IV. For some variables, patient numbers were lower compared to patient numbers in Table II due to censored data. Again, patients with a CA 125 normalisation after two and three courses showed significantly better survival rates. The same was found for t1/2 (d) ⩽ 20 days and t1/2 (d*) ⩽ 16.5 days. Two years survival rates (in %) were significantly better for those patients with CA 125 half-lives below, respectively, 20 days (63%) and 16.5 days (66%).

Finally, a survival analysis was performed, entering all

**Table II** Results of the log rank test on survival curves

| *Variable* | *n* | *M* | $\chi^2$ | *P* | *Variable* | *n* | *M* | $\chi^2$ | *P* |
|---|---|---|---|---|---|---|---|---|---|
| *Stage* | | – | | | *CA 125 III* | | 7 | | |
| III | 37 | | 9.70 | 0.002 | ⩽ $35\,U\,ml^{-1}$ | 21 | | 8.90 | 0.003 |
| IV | 11 | | | | > $35\,U\,ml^{-1}$ | 20 | | | |
| *Histology* | | – | | | *t1/2 (a)* | | 5 | | |
| serous | 30 | | 0.82 | ns | ⩽ 20 days | 27 | | 1.20 | ns |
| rest | 18 | | | | > 20 days | 16 | | | |
| *Grade* | | 2 | | | *t1/2 (b)* | | 5 | | |
| 3 | 37 | | 0.001 | ns | ⩽ 20 days | 29 | | 5.92 | 0.02 |
| 1 + 2 | 9 | | | | > 20 days | 14 | | | |
| *Tumour rest* | | – | | | *t1/2 (c)* | | 2 | | |
| ⩽ 2 cm | 30 | | 3.44 | 0.06 | ⩽ 20 days | 26 | | 5.10 | 0.03 |
| > 2 cm | 18 | | | | > 20 days | 20 | | | |
| *Tumour rest* | | - | | | *t1/2 (d)* | | 16 | | |
| ⩽ 1 cm | 21 | | 5.34 | 0.02 | ⩽ 20 days | 18 | | 8.50 | 0.004 |
| > 1 cm | 27 | | | | > 20 days | 14 | | | |
| *CA 125 I* | | 8 | | | *t1/2 (b*)* | | 5 | | |
| ⩽ $35\,U\,ml^{-1}$ | 8 | | 10.00 | ns | ⩽ 32.6 days | 34 | | 5.01 | 0.03 |
| > $35\,U\,ml^{-1}$ | 32 | | | | > 32.6 days | 9 | | | |
| *CA 125 II* | | 13 | | | *t1/2 (d*)* | | 16 | | |
| ⩽ $35\,U\,ml^{-1}$ | 15 | | 3.82 | 0.05 | ⩽ 16.5 days | 14 | | 7.28 | 0.007 |
| > $35\,U\,ml^{-1}$ | 20 | | | | > 16.5 days | 18 | | | |

*n* = number of patients, M = missing values, ns = not significant.
CA 125 I, II, III: CA 125 levels after respectively one, two and three courses.
t1/2 (a), t1/2 (b), t1/2 (b*) and t1/2 (c): half-life calculations using the formula t1/2 = dt/2log(CA 125-1/CA 125-2).
t1/2 (d) and t1/2 (d*): half-life calculations using the formula CA 125 = exp. [i-s × (days after surgery)].
Half lives marked with an asterisk refer to cut-off levels based on the mean + 2 SD in stage I-II patients.

**Figure 2** Survival curves for patients with a CA 125 normalisation ( $\leqslant 35\ U\ ml^{-1}$) after three courses *vs* patients without a CA 125 normalisation.

**Figure 3** Survival curves for patients with a CA 125 half-life $\leqslant$ 20 days *vs* patients with a CA 125 half-life > 20 days. Half-lives [t1/2 (d)] were calculated using the formula: ln [serum CA 125 = i-s (days after surgery)].

significant prognostic variables as covariates in a Cox Proportional Hazards model. Stage of disease provided the best correlation with overall survival ($P = 0.006$). Of all other variables which showed a significant correlation with survival in the univariate analysis (Table II), only t1/2 (d) could further improve survival correlation ($P = 0.04$). Thus, the calculated half-life according to the formula of Buller *et al.* using a cut-off of 20 days, was the only additional independent prognostic factor for survival in addition to FIGO stage.

### Discussion

In this study the prognostic value of early CA 125 regression following cytoreductive surgery and during first-line

**Table III** CA 125 half-lives (in days) in stage I–II ovarian cancer patients

| | *n* | *Mean* | *SD* | *Cut-off* |
|---|---|---|---|---|
| t1/2 (a) | 9 | 10.7 | 6.16 | 23 |
| t1/2 (b) | 9 | 14.4 | 9.12 | 32.6 |
| t1/2 (c) | 11 | 36.5 | 38.99 | 112.5 |
| t1/2 (d) | 5 | 9.8 | 3.34 | 16.5 |

Mean, standard deviation (SD) and derived cut-off values (mean + 2SD) are listed. *n* = number of patients for whom a half-life could be calculated. t1/2 (a), t1/2 (b) and t1/2 (c): half-life calculations using the formula: t1/2 = dt/2log (CA 125-1/CA 125-2). t1/2 (d): half-life calculated using the formula: CA 125 = exp. [i-s × (days after surgery)]. Serum levels used for half-life calculations are specified in the text.

chemotherapy in ovarian cancer was assessed. Patients with stage III–IV ovarian cancer and a CA 125 normalisation after three courses of chemotherapy and those with a CA 125 half-life ⩽ 20 days had a significant better prognosis than patients who had not normalised after the third course or who had a CA 125 half-life > 20 days. This is in accordance with previous reports (Lavin *et al.*, 1987; Sevelda *et al.*, 1989; Mogensen *et al.*, 1990; Redman *et al.*, 1990).

In one study, the risk of dying of ovarian cancer for those patients with elevated CA 125 levels 3 months after surgery was three times as high as for patients whose serum levels had normalised (Sevelda *et al.*, 1989). However, a CA 125 normalisation remains of limited predictive value for the definitive chance of cure, as Mogensen *et al.* reported that 23 out of 47 patients with residual tumour at second-look had shown normal CA 125 serum levels after three courses of chemotherapy (Mogensen *et al.*, 1990). In the present study, 5 out of 17 stage III–IV patients died within 2 years after primary surgery despite a CA 125 normalisation after three courses of chemotherapy.

Mogensen subdivided patients according to absolute CA 125 serum levels after three courses of chemotherapy. Significantly different survival rates were observed for patients with CA 125 levels ⩽ 10 U ml$^{-1}$, 11–100 U ml$^{-1}$ and > 100 U ml$^{-1}$ independently of other prognostic factors (Mogensen, 1992). However, even in the group with CA 125 values ⩽ 10 U ml$^{-1}$, 50% died within 5 years after surgery. In his study, Mogensen used both the Abbott RIA and Abbott EIA. Reported CA 125 levels will be different when using the original Centocor CA 125 RIA since there are considerable differences between the various assays available (Yedema *et al.*, 1992*a*).

As shown recently, the time interval within which mean CA 125 serum levels differ significantly between therapy responders and non-responders can be as short as 1 to 4 weeks after surgery (Cruickshank *et al.*, 1992). However, within the group of patients with residual disease > 2 cm, a discrimination between responders and non-responders could not be made that early.

Using pre-chemotherapy CA 125 baseline levels, CA 125 half-life values have been reported to be inversely related to the duration of progression free survival and overall survival (Van der Burg *et al.*, 1988; Hawkins *et al.*, 1989; Hogberg & Kagedal, 1990). This observation could not be confirmed in our study. The timespan between surgery and moment of sampling may have influenced results in our study and those reported by others. Cytoreductive surgery itself may cause a transient CA 125 rise (Yedema *et al.*, 1993). This effect can last for at least 2 weeks and is predominantly seen if pre-surgery levels are relatively low. As a result, if CA 125 sampling has been performed shortly after laparotomy, this baseline value may not be a reliable parameter. Median time between surgery and pre-chemotherapy sampling was 14 days in our study group. No exact data concerning this aspect are available from other studies (Hawkins *et al.*, 1989, Hogberg & Kagedal, 1990; Van der Burg *et al.*, 1988). Furthermore, patient selection may explain these differences. Once enrolled in the present study, no patients were excluded while others only included patients showing a favourable response to therapy (Hawkins *et al.*, 1989; Hogberg & Kagedal, 1990) thus excluding patients with progressive or stable disease. While we studied only stage III–IV patients, Van der Burg included a large portion of stage I–II patients in her study group (Van der Burg *et al.*, 1988). These patients will have a low progression rate and prolonged survival and CA 125 is expected to normalise within a short time once chemotherapy is initiated. Consequently, patients with early stage I–II disease will be over-represented in the short half-life group.

We found a significant correlation between CA 125 half-life and survival only if pre-surgery levels were taken as a baseline value. These data are in agreement with those of others using pre-surgery baseline values (Hunter *et al.*, 1990; Willemse *et al.*, 1991).

The best correlation between the CA 125 half-life and survival was provided using the exponential regression model of Buller *et al.* (Buller *et al.*, 1991). In their study, CA 125 half-life predicted progressive disease and lack of respon-

**Table IV** Significant prognostic variables and survival percentages at 2 years follow-up

| *Variable* | *n* | *deaths* | *% survival* | *P* | *Odds ratio* | *CI* |
|---|---|---|---|---|---|---|
| *Stage* | | | | | | |
| III | 33 | 17 | 0.45 | 0.03 | 8.47 | (0.96–74.6) |
| IV | 10 | 9 | 0.10 | | | |
| *Tumour rest* | | | | | | |
| ⩽ 1 cm | 18 | 7 | 0.61 | 0.02 | 4.98 | (1.33–18.6) |
| > 1 cm | 25 | 19 | 0.24 | | | |
| *CA 125 II* | | | | | | |
| ⩽ 35 U ml$^{-1}$ | 12 | 4 | 0.67 | 0.03 | 5.60 | (1.16–27.1) |
| > 35 U ml$^{-1}$ | 19 | 14 | 0.26 | | | |
| *CA 125 III* | | | | | | |
| ⩽ 35 U ml$^{-1}$ | 17 | 5 | 0.71 | 0.001 | 12.80 | (2.55–64.4) |
| > 35 U ml$^{-1}$ | 19 | 16 | 0.16 | | | |
| *t1/2 (d)* | | | | | | |
| ⩽ 20 days | 16 | 6 | 0.63 | 0.01 | 9.17 | (1.49–56.3) |
| > 20 days | 13 | 11 | 0.15 | | | |
| *t1/2 (d*)* | | | | | | |
| ⩽ 16.5 days | 12 | 4 | 0.66 | 0.01 | 9.33 | (1.65–52.6) |
| > 16.5 days | 17 | 14 | 0.18 | | | |

*n* = number of patients. Odds ratios and 95% confidence intervals (CI) are included CA 125 II, III: CA 125 levels after respectively two and three courses. t1/2 (d) and t1/2 (d*): half-life calculations using the formula: CA 125 = exp. [i-s × (days after surgery)]. The half-life marked with an asterisk refers to a cut-off based on the mean + 2SD in stage I–II patients (Table III). Serum levels used for half-life calculations are specified in the text.

**Figure 4** CA 125 half-life [t1/2 (d)] and survival in stage III–IV patients. Closed circles symbolise deceased patients. Open circles symbolise survivors.

siveness to chemotherapy. In our multiple regression analysis this factor was, in addition to stage of disease, the only independent prognostic variable in predicting survival. Thus, our data show that this model can be used as an independent predictor of survival.

The half-life of CA 125 after complete debulking is a particular point of interest. Canney *et al.* found a CA 125 half-life of 4.8 days based on one single observation (Canney *et al.*, 1984). Buller *et al.* reported a mean serum half-life of 10.8 days (Buller *et al.*, 1991) in their study population, which included a substantial portion of completely resected stage III–IV patients, some of whom might suffer from early tumour recurrence. This occurred in two stage III patients from the present series at 9 and 18 months after complete cytoreductive surgery. All stage I–II patients in the present study who underwent complete cytoreductive surgery were without evidence of disease in the follow-up period (median 29 months, range 8–85). In these 12 patients, the mean CA 125 half-life t1/2 (d) according to Buller was 9.8 days. At a cut-off of 16.5 days (mean + 2SD) univariate analysis showed a significant correlation with survival, while in a multivariate model, no independent prognostic value could be found ($P = 0.09$). We believe that CA 125 regression in stage I–II patients deserves further attention to obtain an estimate of the ideal CA 125 regression curve which could serve as a reference for patients with advanced disease.

In the present study there were short term and long term survivors in respectively the short CA 125 half-life ($\leqslant 20$ days) and the long CA 125 half-life group ($>20$ days) (Figure 4). Also in previous studies based on absolute CA 125 values, overlapping values between good and poor responders were frequently found. The CA 125 half-life based on the exponential regression model according to Buller was the only variable with a significant prognostic value other than stage in the multivariate analysis. Whether or not the CA 125 half-life based on serial measurements early during therapy is a better discriminator between good and poor responders than single CA 125 measurements before therapy and after three courses remains to be elucidated. A large scale multi-centre study might give the answer which CA 125 response at what stage during therapy provides the best estimation of the survival time to be expected.

The authors are indebted to C.M.G. Thomas PhD. (Department of Obstetrics and Gynecology, University Hospital Nijmegen, Nijmegen, the Netherlands) and to G.J. van Kamp PhD. (Department of Clinical Chemistry, Free University Hospital, Amsterdam, the Netherlands) for their laboratory work. We thank A.J. de Grient Dreux and K. Hudson for preparation of the manuscript. This study was in part supported by The Dutch Praeventie Fonds, grant No. 28-1248.

### References

BAST, R.C., KLUG, T.L., ST JOHN, E., JENISON, E., NILOFF, J.M., LAZARUS, H., BERKOWITZ, R.S., LEAVITT, T., GRIFFITHS, T., PARKER, L., ZURAWSKI, V.R. & KNAPP, R.C. (1983). A radioimmunoassay using a monoclonal antibody to monitor the course of epithelial ovarian cancer. *N. Engl. J. Med.*, **309**, 883–887.

BULLER, R.E., BERMAN, M.L., BLOSS, J.D., MANETTA, A. & DISAIA, P.J. (1991). CA 125 regression: a model for epithelial ovarian cancer response. *Am. J. Obstet. Gynecol.*, **165**, 360–367.

VAN DER BURG, M.E.L., LAMMES, F.B., VAN PUTTEN, W.L.J. & STOTER, G. (1988). Ovarian cancer: the prognostic value of the serum half-life of CA 125 during induction chemotherapy. *Gynecol. Oncol.*, **30**, 307–312.

CANNEY, P.A., MOORE, M., WILKINSON, P.M. & JAMES, R.D. (1984). Ovarian cancer antigen CA 125: A prospective clinical assessment of its role as a tumour marker. *Br. J. Cancer*, **50**, 765–769.

CRUICKSHANK, D.J., TERRY, P.B. & FULLERTON, W.T. (1992). CA 125 response assessment in epithelial ovarian cancer. *Int. J. Cancer*, **51**, 58–61.

DIXON, W.J. (ed). (1983). *BMDP Statistical Software*. Berkeley University of California Press.

HAWKINS, R.E., ROBERTS, K., WILTSHAW, E., MUNDY, J., FRYATT, I.J. & MCCREADY, V.R. (1989). The prognostic significance of the half-life of serum CA 125 in patients responding to chemotherapy for epithelial ovarian carcinoma. *Br. J. Obstet. Gynaecol.*, **96**, 1395–1399.

HOGBERG, T. & KAGEDAL, B. (1990). Serum half-life of the tumor marker CA 125 during induction chemotherapy as a prognostic indicator for survival in ovarian carcinoma. *Acta Obstet. Gynecol. Scand.*, **69**, 423–429.

HUNTER, V.J., DALY, L., HELMS, M., SOPER, J.T., BERCHUCK, A., CLARKE-PEARSON, D.L. & BAST, R.C. (1990). The prognostic significance of CA 125 half-life in patients with ovarian cancer who received primary chemotherapy after surgical cytoreduction. *Am. J. Obstet. Gynecol.*, **163**, 1164–1167.

JACOBS, I. & BAST, R.C. (1989). The CA 125 tumour-associated antigen: a review of the literature. *Hum. Reprod.*, **4**, 1–12.

KENEMANS, P., BAST, R.C., YEDEMA, C.A., PRICE, M.R. & HILGERS, J. (1988). CA 125 and polymorphic epithelial mucin as serum tumor markers. *Cancer Rev.*, **11/12,** 119–144.

KOTTMEIER, H.L. (1976). FIGO staging system. *Gynecol. Oncol.*, **4,** 13–19.

LAVIN, P.T., KNAPP, R.C., MALKASIAN, G., WHITNEY, C.W., BEREK, J.C. & BAST, R.C. (1987). CA 125 for the monitoring of ovarian carcinoma during primary therapy. *Obstet. Gynecol.*, **69,** 223–227.

MOGENSEN, O., MOGENSEN, B., JAKOBSEN, A. (1990). Predictive value of CA 125 during early chemotherapy of advanced ovarian cancer. *Gynecol. Oncol.*, **37,** 44–46.

MOGENSEN, O. (1992). Prognostic value of CA 125 in advanced ovarian cancer. *Gynecol. Oncol.*, **44,** 207–212.

REDMAN, C.W.E., BLACKLEDGE, J.R., KELLY, K., POWELL, J., BUXTON, E.J. & LUESLY, D.M. (1990). Early serum CA 125 response and outcome in epithelial ovarian cancer. *Eur. J. Cancer*, **26,** 593–596.

RUSTIN, G.J.S., GENNINGS, J.N., NELSTROP, A.E., COVARRUBIAS, H., LAMBERT, H.E. & BAGSHAWE, K.D. (1989). Use of CA 125 to predict survival of patients with ovarian carcinoma. *J. Clin. Oncol.*, **7,** 1667–1671.

SEVELDA, P., SCHEMPER, M., SPONA, J. (1989). CA 125 as an independent prognostic factor for survival in patients with epithelial ovarian cancer. *Am. J. Obstet. Gynecol.*, **161,** 1213–1216.

WILLEMSE, P.H.B., AALDERS, J.G., DE BRUYN, H.G.A., MULDER, N.H., SLEIJFER, D.TH. & DE VRIES, E.G.E. (1991). CA 125 in ovarian cancer: Relation between half-life, doubling time and survival. *Eur. J. Cancer*, **27,** 993–995.

YEDEMA, C.A., THOMAS, C.M.G., SEGERS, M.F.G., DOESBURG, W.H. & KENEMANS, P. (1992*a*). Comparison of five immunoassay procedures for the ovarian carcinoma-associated antigenic determinant CA 125 in serum. *Eur. J. Obstet. Gynecol. Reprod. Biol.*, **47,** 245–251.

YEDEMA, C.A., KENEMANS, P., THOMAS, C.M.G., MASSUGER, L.F.A.G., WOBBES, TH., VERSTRAETEN, A.A., VAN KAMP, G.J. & HILGERS, J. (1993). CA 125 serum levels in the early post-operative period do not reflect the outcome of cytoreductive surgery. *Eur. J. Cancer*. (in press).